# Biopsychosocial impact of keloids on quality of life

**DOI:** 10.1016/j.jdrv.2024.08.010

**Published:** 2024-08-23

**Authors:** Megan Mathew, Gabrielle DiBartolomeo, Rivka C. Stone

**Affiliations:** a Department of Dermatology, UT Southwestern Medical Center, Dallas, Texas; b Departmentof Internal Medicine, Cleveland Clinic Florida, Weston, Florida; c Dr.Phillip Frost Department of Dermatology and Cutaneous Surgery, University of Miami Miller School of Medicine, Miami, Florida

**Keywords:** dermatology life quality index, keloids, psychosocial well-being, quality of life, scars, skin of color

## Abstract

Keloids are a prevalent, although understudied, dermatologic condition commonly affecting people of color. In the clinical setting, keloids negatively impact many aspects of patient quality of life (QoL), but this area has not been comprehensively characterized. This review identifies and summarizes studies in which the QoL impact of keloids has been measured utilizing validated survey and questionnaire instruments, including the SCAR-Q and Dermatology Life Quality Index. Study findings are grouped into physical and emotional factors that together comprise 7 biopsychosocial domains: pain and itch, mobility, visibility, gender and age, psychosocial, stigma, and cosmetic. These domains interact in a complex network that negatively impacts QoL of patients with keloid. Enhanced understanding of the significance of these domains will pave the way for the performance of large, rigorous epidemiologic studies that will lead to the development of efficacious keloid therapies that improve not only physical but also psychosocial functioning, enabling clinicians to provide holistic care that enhances patient QoL.

## CAPSULE SUMMARY

- This review comprehensively synthesizes studies of keloids and their impact on quality of life through the lens of 7 biopsychosocial themes.
- Quality of life assessments should be incorporated into the evaluation and management of all keloid patients to provide optimal holistic treatment.

## INTRODUCTION

Keloids are a benign dermal fibroproliferative disorder resulting from an abnormal wound healing response.^1,2^ Although their pathophysiology is not fully elucidated, keloids form due to prolonged inflammation, leading to fibroblast proliferation and excess collagen production.^2,3^ The development of keloids is multifactorial, involving environmental triggers and genetic predisposition.^3^ Although they may develop spontaneously, most keloids arise from sites of trauma, including the earlobes after piercing, the extremities after lacerations or burns, and postacne keloidal scars.^1,2^

Clinically, keloids are firm, raised, smooth proliferations of scar tissue that can present in a variety of colors and sizes.^1^ In contrast to hypertrophic scars, keloids extend beyond the boundary of the original wound, are unlikely to spontaneously regress, and may continue to grow years after the initial insult.^1,3,4^ Keloids are present across all ethnicities but affect darker-skinned individuals at a higher rate, with an incidence of 4% to 16% in Black and Hispanic people.^5,6^ Black and Asian populations also have a more severe keloid phenotype in terms of size and number of lesions.^7^ Although keloids are one of the most prevalent dermatologic diseases, they are relatively understudied.^8^ Keloids have been receiving more attention recently, along with growth in the body of skin and color research.^1,2,6,9^ Yet, a major understudied feature of keloids is their impact on patients’ quality of life (QoL).^10^ Skin diseases as a whole negatively affect QoL to a degree comparable with or exceeding that of life-threatening illnesses, such as myocardial infarction or heart failure,^11^ and studies have shown that QoL deterioration attributed to keloids is comparable with that of patients with chronic skin conditions such as psoriasis.^1,9,10,12,13^ Keloids are challenging to treat not only because there is no gold standard for treatment^14^ but also because current interventions still carry a high risk of recurrence.^1^

As more studies are performed to develop better treatment options for keloids,^10^ we believe that it is just as important to shed light on the significant impact keloids have on patients’ QoL. Keloid therapies are often considered cosmetic and are not covered by insurance. As such, by highlighting the significant negative impact of keloids on QoL, we can better advocate for expanded coverage of treatments that target QoL across biopsychosocial domains. Furthermore, enhanced understanding of keloid impact on QoL by clinicians and researchers will encourage holistic patient-centered care and drive the development of efficacious therapies that improve patient lives.

## QUALITY OF LIFE ASSESSMENT IN PATIENT WITH KELOID POPULATIONS

We searched Embase and MEDLINE databases for studies evaluating the QoL impact of keloids using the search terms “keloid” or “keloid disease” and “quality of life assessment,” “quality of life,” or “psychological rating scale.” We screened 340 unique results to identify 17 original studies that directly assessed the impact of keloids on keloid-associated QoL using scale- or questionnaire-based instruments. We excluded studies not performed in keloid patient cohorts and/or not incorporating QoL measurement, as well as interventional studies missing a nonkeloid control group (Supplementary Fig 1 and Supplementary Table I, available via Mendeley at https://doi.org/10.17632/7bstmt69p3.1). Many studies used dermatologic QoL instruments developed for other skin diseases,^15^ making it difficult to rank instruments by validity and reproducibility in patients with keloids. Four of the studies that met inclusion criteria used the Dermatology Life Quality Index^11,12,16,17^ suggesting its validity and reproducibility in patients with keloids. Several instruments (Skindex-29, 36-Item Short Form Survey [SF-36], and 5 Level EuroQuol 5 Dimension Questionnaire [EQ-5D-5L]) were noted to be concordant with each other, suggesting that they accurately capture a keloid QoL phenotype.^9^ The remaining studies created new questionnaires and/or conducted semistructured interviews to capture subjective patient experiences in particular QoL domains, with an associated risk of bias.^11,18^

We incorporated findings from these 17 studies with complementary reports from the literature to comprehensively characterize how keloids impact patient QoL through 7 novel biopsychosocial domains: pain and itching, mobility, visibility, gender and age, psychosocial, stigma, and cosmetic (Table I).^4,11,13,14,19–22^ These global themes provide a new way to capture the breadth of keloids’ impact on the physical and emotional well-being of patients.

### Physical factors

Physical factors constitute a major area of impact on QoL in patients with keloids. These factors include pain and itch, mobility, visibility, and gender and age.

### Pain and pruritus

Pain and pruritus are common clinical features of keloids.^1,20–22^ Unsurprisingly, a majority of the studies found that pain and pruritus negatively impacted patients’ QoL.^9,11,16–18,19,23–30^ Several studies found that these 2 factors were the most consistent indicators of impaired health-related QoL.^9,27,29,30^ This is supported by the fact that pain was found to be one of the top reasons patients choose to seek medical care for their keloids.^31^ Additionally, both pain and pruritus were found to directly hinder daily tasks.^11^ Hyperpigmented keloids or those patients with keloids for ≥10 years were more likely to report associated pain and itch.^25,30^ Additionally, symptomatic keloids refractory to treatment negatively impacted QoL.^30^ As such, finding additional effective treatment options to manage keloid-associated pain and pruritus is critical to improving QoL.

Contributing to the decreased QoL could be the fact that chronic itch conditions are associated with higher rates of stress, anxiety, depression, and, in some severe cases, can lead to suicidal ideation.^32,33^ Further complicating the matter, psychological and emotional factors have been shown to increase the perception of itch, leading to worsening symptoms and suboptimal treatment outcomes.^32^ A recent study even suggested that psychological stress may play a role in the pathogenesis of keloids.^34^

### Mobility

Many of the keloid QoL measurement tools assessed the impact of keloids on joint mobility. Keloids, particularly larger lesions that involve the shoulders, knees, and elbows, may affect the mobility of major joints.^35^ However, even lesions involving the fingers, toes, back, and chest can affect mobility by creating the sensation of stretching, reducing range of motion, and triggering pain with movement. These restrictions were found to interfere with the ability of patients to complete everyday tasks, negatively impacting their QoL.^23,24,30,36^

These studies and the questionnaires used focus on the impact the keloid directly has on joint mobility. Interestingly, a recent orthopedic study found that patients with a history of keloids are at a higher risk of developing arthrofibrosis, a postoperative complication requiring readmission and surgical revision.^37^ As more studies are performed to explore the link between keloids and other systemic conditions,^8^ we can better appreciate the impact of keloids on QoL.

### Visibility

Keloids have been reported to affect most parts of the body, including areas not easily concealed by clothing. Surprisingly, keloids located in visible areas were not consistently associated with a globally reduced QoL.^9,23,36,38^ With that said, visible lesions were associated with higher subjective scores of relationship sensitivity (feeling uncomfortable with the opposite gender, feelings of inferiority, and concerns of being criticized), depression, and anxiety than nonvisible keloids,^36,38^ in part due to the perceived greater need for concealment strategies and coping mechanisms.^16,18,25^

### Age and gender

Although keloids may affect patients of all ages, lesions are more frequently present in younger patients aged between 10 and 30 years,^1,3,39^ which was reflected in the demographics of patients enrolled in keloid QoL studies.^12,16–18,24,26,36^ In one study, younger patients reported more significant reductions in QoL than older patients, with the highest negative impact on QoL noted between ages 16 and 35 years.^16,36^ Contributing to the decreased QoL, one study found an association with the recurrence of keloids and younger age.^30^ Presently, there is no study investigating the longitudinal impact on QoL in these patients.

With respect to gender, several studies found no significant difference in keloid QoL impairment between men and women.^19,23,26,40^ Another study found that women undergoing surgical treatment for keloids experienced more preoperative pruritus than men.^19^ However, enrolled women largely outnumbered men in many of the study populations, which may have skewed these findings.^11,16,19,26,40^ The transgender patient population faces unique QoL considerations, as keloids forming near operative sites of gender-affirming surgery can contribute to gender dysphoria.^41^ Similarly, facial keloids in individuals with pseudofolliculitis barbae have a negative impact on transgender identity.^41,42^ Additional studies are needed to fully assess the impact on QoL in this population.

## EMOTIONAL WELL-BEING

The impact of keloids on patients’ emotional well-being is another significant source of QoL impairment. These factors are divided into the psychosocial, stigma, and cosmetic domains.

### Psychosocial

Scarring–of which keloids are considered a subtypedhas been found to negatively impact body image, contribute to body dysmorphic disorders, to negatively impact social development, and to be associated with the development of mental health disorders.^10,13^ It is not surprising then that the studies found the psychosocial impacts of keloids are extensive and contribute heavily to the QoL impairments reported by patients.^9,16,17,23,24,27,36^ One study found that nearly half of patients experienced severe emotional symptoms,^27^ whereas another study specifically identified higher levels of feelings of shame.^25^ Keloids affect interpersonal relationships and hinder patients’ abilities to interact socially with others, because individuals experience embarrassment and anxiety regarding their condition.^17^ The stigma associated with keloids leads to social impairment and resultant depression and anxiety.^36^ One study further identified higher levels of depression and anxiety in patients who had more visible keloids.^36^ Patients also reported a negative impact on sexual health in association with feelings of selfconsciousness when keloids were in sensitive areas.^24^

A study compared 2 cohorts of patients with keloids, with one group of patients screening positive for symptoms consistent with body dysmorphic disorder and a control group that did not report these symptoms. Interestingly, there were no distinguishing factors, such as age, gender, keloid location, clinical symptoms (such as pain or pruritus), or etiology (such as piercings, acne, or surgery), between the 2 groups.^13^ This suggests that developing a negative body image is patient-specific and not something that can be objectively determined based on keloid symptoms and characteristics. This further indicates the importance of screening each patient with keloids regardless of severity to address the impact on their QoL, including their mental health.

In 2 interview-based studies, patients described qualitative feelings of unhappiness and stigma that led to coping behaviors to hide or compensate for their keloids.^11,18^ In these studies, patient-reported experience with medical care and treatment of their keloids influenced psychosocial impact. Uncertainty regarding diagnosis, concerns about treatment efficacy, and a lack of empathy from physicians further amplified patient distress.^11^ On the other hand, successful treatment of keloids resolved feelings of distress and embarrassment,^19^ improving QoL in the psychosocial domain.

### Stigma

Patients with many dermatologic diseases often report “stigmatization,” as defined by being judged, discriminated against, or disapproved of by others due to their skin condition.^36^ Several studies reported that patients with keloids often believed stigmatized or experienced emotional distress due to a fear of experiencing stigma.^16,18,24,36,40,43^ Specifically, patients expressed concerns that others associated the presence of keloids with a history of criminal activity and/or with self-inflicted injuries.^16^ In one study, as many as 45% of participants reported unwanted attention because of their keloids.^24^ That said, stigmatization is not universally experienced and may vary based on cultural norms. As an example, one study conducted in Nigeria found no impact on QoL by keloids in patients of African descent.^11,40^ This may be explained by their endemic nature; in fact, keloids are recognized as part of tribal culture and considered an attribute of beauty.^11,40^

### Cosmetic

The cosmetic features of keloids include color, thickness, and texture. Keloids can appear purple or pink, skin colored, hyperpigmented, or hypopigmented.^44^ The degree of vascularity also contributes to overall appearance. Keloids vary in thickness as well as texture, ranging from pliable to rigid, with irregular or straight margins.

Of the included studies, only 2 objectively investigated how keloid pigmentation may affect QoL. Although one study did not find a clear relationship between coloration and QoL,^9^ the other study found hyperpigmented keloids were associated with decreased QoL.^30^ However, this may be confounded by the observation that hyperpigmented keloids were significantly more likely to be painful, which in and of itself negatively impacts QoL. Keloid irregularity and pliability were found to be less impactful on QoL as compared with physical factors such as pain and pruritus.^9,16,19,27^

## OVERLAP OF BIOPSYCHOSOCIAL THEMES

**Fig 1.**
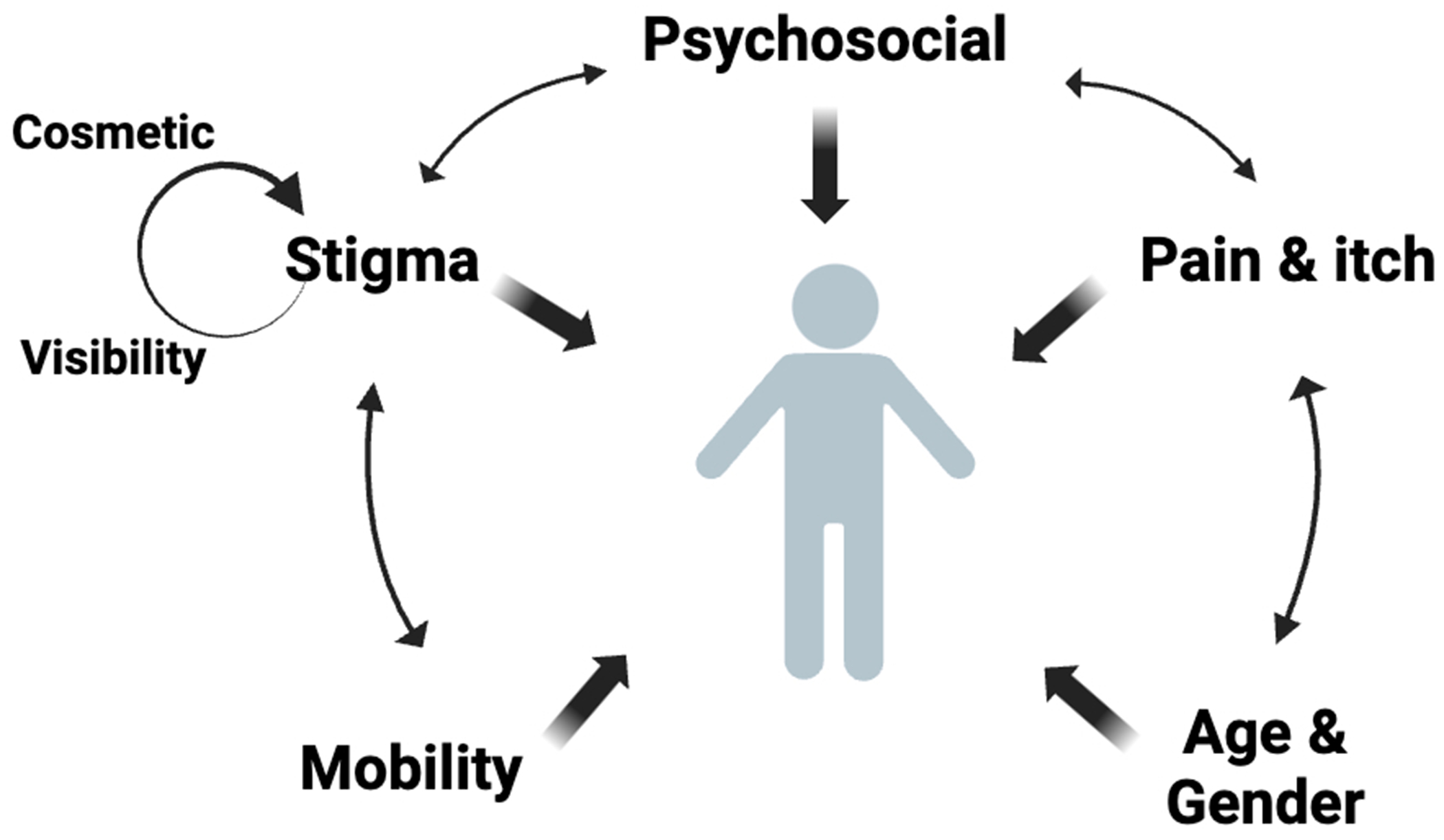
Overlapping domains affecting quality of life in patients with keloid.

In this review, we have separated the impact of keloids on QoL into 7 domains that encompass physical and emotional factors. However, studies recognize that these domains do not function in isolation; rather, their interplay further contributes to a negative global QoL impact (Fig 1). For instance, physical factors affect emotional well-being, because patients experiencing high degrees of pain and itch were found to have severe emotional health related QoL impairment as a result.^9^ Similarly, psychological factors such as dissatisfaction with appearance and feelings of embarrassment were positively correlated with physical factors such as pruritus, pain, and restriction.^25^ Personal stigma associated with keloids generates a negative psychosocial impact, which further perpetuates societal stigma. On the other hand, lower levels of keloid-associated societal stigma can lessen the negative psychosocial impact on QoL and improve overall emotional well-being.^40^

## CONCLUSIONS AND FUTURE DIRECTIONS

Keloids affect patients’ physical health, emotional well-being, and social interactions. As demonstrated in this review, the domains of pain and itch, mobility, visibility, gender and age, psychosocial, stigma, and cosmetic, reflect the impact of keloids on patients’ lives and overall well-being, and the interplay of these factors multiplies keloid disease burden. Despite the scope and significance of these effects, most keloid research focuses on treatment, and QoL improvement remains significantly understudied. When viewed by society and by policymakers as a purely cosmetic condition, keloids may be deemed of lower priority not only for research studies,^27,45^ but also may not be reimbursed by insurers as a medical condition. By shifting the context and focusing on improving QoL metrics, health care professionals can raise the priority of keloids in health policy decision-making, allowing higher-quality treatment options and easier access to patient resources.

Given the paucity and limitations of keloid QoL research to date, larger epidemiologic studies in more diverse patient populations are needed. However, this review underscores the need to prioritize the development of robust QoL instruments in the keloid population that are not only valid and reproducible across patient phenotypes (age, gender, race/ethnicity, keloid location, etc), but also accurately capture the spectrum of biopsychosocial factors that impact QoL in these patients. Standardization of future studies to utilize these validated keloid-specific QoL instruments will provide data to support the expansion of insurer coverage for keloid treatments as medical conditions. In addition, these studies will hopefully drive the development of novel therapeutics specifically targeting improvement in QoL metrics, with a significant impact on patient lives. Finally, a better understanding of the economic impact of poor keloid-associated QoL is warranted, especially because keloids are often refractory to treatment and pose a chronic burden to the health care system. With increased awareness of the negative impact of keloids on QoL, clinicians will be able to refine their treatment approaches to holistically improve patient lives across the biopsychosocial domains.

## Supplementary Material

Supplemental Table 1

Supplementary Figure 1

## Figures and Tables

**Table I.** Assessment of biopsychosocial domains impacting quality of life of patients with keloid

Themes	Assessment tools	Examples of question items	References
Pain and itch	Skindex-29 SF-36 QualiFibro DLQI Interviews SCAR-Q EQ-5D-5L	• Frequency of skin hurting or itching • Severity of pain • Frequency of reduced sleep • Frequency of skin burning or stinging • Difficulty working	^4,11,13,14,19^
Mobility	QualiFibro DLQI SCL-90 Interviews EQ-5D-5L	• Difficulty level in walking • “How bothered have you been in the last week by muscle soreness?” • Limitations in playing sports	^14,20^
Visibility	Skindex-29 SF-36 QualiFibro DLQI Interviews SCL-90	• “How often has skin influenced your clothing?” • Feeling uneasy when others are watching you	^20–22^
Gender and age	Skindex-29 DLQI Interviews SCAR-Q	• “How old are you?” • “What is your gender?”	^14,19,21,22^
Psychosocial	Skindex-29 SF-36 QualiFibro DLQI Interviews SCL-90 EQ-5D-5L SCAR-Q	• Frequency of needing to stay home • Frequency of skin condition affecting interactions with others • Skin condition affecting desire to be with people • Frequency of being annoyed with skin condition • Less time spent on activities such as shopping or gardening • Condition affecting sexual activity • Frequency of depressive symptoms • “How much energy do you have?”	^11,13,19–21^
Stigma	DLQI Interviews SCL-90	• Frequency of feeling embarrassed or self-conscious • Frequency of feeling like others do not like you • Feeling inferior to others • Feeling you are being watched or talked about	^14,20,22^
Cosmetic	Skindex-29 SF-36 DLQI EQ-5D-5L SCAR-Q POSAS	• Rating severity of wound features from 1 (normal) to 10 (worse scar imaginable)	^19,22^

*DLQI*, Dermatology Life Quality Index; *EQ-5D-5L*, 5 Level EuroQuol 5 Dimension Questionnaire; *POSAS*, The Patient and Observer Scar Assessment Scale; *SCL-90*, Symptom Checklist 90; *SF-36*, 36-Item Short Form Survey.

## Abbreviation used:

QoL: quality of life
