# Supplementary material for: Biopsychosocial impact of keloids on quality of life
Source: JAAD Rev. Author manuscript; Available in PMC 2025 Aug 1. (PMC12314879; doi:10.1016/j.jdrv.2024.08.010)
Supplement: Supplemental Table 1 [file NIHMS2095636-supplement-Supplemental_Table_1.docx]

| **Supplemental Table A: Summary of Original Articles assessing the Biopsychosocial Impact of Keloids on Quality of Life (QoL)** | | | | | |
| --- | --- | --- | --- | --- | --- |
| **Study**  **Reference** | **Study**  **Design** | **Number of Keloid**  **Patients** | **Setting** | **QoL Tools Used** | **Key Findings** |
| Balci et al., 2009 ​[12] | Prospective case control | 48 | Antaya, Turkey | DLQI | - Total DLQI scores of patients with keloids and hypertrophic scarring (HTS) (7.79 ± 5.10) and psoriasis (8.73 ± 5.63) were comparable and significantly higher than that of healthy controls (0.58 ± 0.77). - “Symptoms and feelings” subscale had the highest score in both keloids/hypertrophic scarring and psoriasis groups. - There was no significant difference in scores between keloids and HTS groups vs. psoriasis group. |
| Furtado et al., 2009 ​[24]​ | Cross-  sectional | 102 | Sao Paulo, Brazil | QualiFibro | - Higher physical severity in patients with keloids in non-visible areas (p < 0.01). - Higher physical severity in patients with keloids for longer than 10 years duration (p < 0.01). - Psychological factors (lack of satisfaction about appearance and feelings of embarrassment) positively correlated with physical factors (pruritus, pain, movement restriction). |
| Bijlard et al., 2017 ​[9]​ | Cross-  sectional | 106 | Rotterdam, Netherlands | POSAS, Skindex-29, SF-36, EQ-5D-5L | - Keloid patients reported a worse mental HRQL. - Pain and itch were associated with nearly all HRQL measures (p <0.001). - Scar stiffness, thickness, and irregularity showed high correlations with HRQL outcomes. - Female sex correlated with worse outcomes on emotional, symptomatic, and sum scores (p <0.017). - 48% had severe emotional symptoms and about a quarter reported severe problems on the symptomatic and functional scale. - Keloid patients scored significantly lower on the dimensions of bodily pain, vitality, and social functioning as well as on the mental component summary. - Itching and painful keloids were associated with the largest HRQL impairment, while cosmetic factors (color, thickness, pliability, and irregularity) were less related. |
| Brown et al., 2008 ​[11]​ | Qualitative Interview Study | 34 | Manchester, United  Kingdom | One-to-one semi-structured interviews | - 56% were dissatisfied and concerned about perceived stigma and psychological associations others may make regarding their keloids. - Found that some adopted coping behaviors to hide keloids. - 21% felt their keloids interfered with communication skills and personal relationships. |
| Hsieh et al., 2021 ​[18]​ | Qualitative Interview Study | 37 | Chicago, USA | Semi-structured interviews | - Eight themes: (1) psychological well-being, (2) social well-being, (3) attempts to conceal, (4) determinants of opinion of scar, (5) sexual well-being, (6) health/physical well-being, (7) career, and (8) overall satisfaction of scar - Patients spoke commonly about psychological and social well-being (i.e. frequency of thinking about a scar and talking about scars with others). - Discussions of sexual well-being and career were elicited but rarer (i.e. feeling uncomfortable when naked and negative impacts on professional networking). |
| Guy et al., 2025 [31] | Prospective cohort | 40 | Houston, USA | Head and neck keloid QoL questionnaire | - Developed questionnaire specific for head & neck keloids evaluating impact on QoL in four main domains: physical symptoms, self-esteem, social functioning and medical motivation. - The majority of patients reported seeking medical care due to physical concerns including pain, growth and change in appearance. - The physical symptom subscale had the strongest correlation to the overall impact on QoL, while medical motivation had the weakest correlation. |
| Bock et al., 2006 ​[22]​ | Cross-  sectional | 100 | Kiel, Germany | Custom questionnaire | - Correlation of physical impairment with pain (p ≤0.001), pruritus (p <0.001), and the amount of restriction of mobility (p <0.001). - Psychological scale was associated with pain and restriction of mobility. - The impairment of QoL in patients with keloids and HTS is not only caused by their skin symptoms but also by the psychological aspects due to stigmatization. |
| Kassi et al., 2020 ​[16]​ | Cross-  sectional | 132 | Abidjan, Ivory Coast | DLQI | - Moderate to high DLQI scores in 61.66% of patients. - QoL impacted significantly when keloid was associated with pain (p =0.046), pruritus (p =0.81) and functional disorders (p =0.29). - QoL was impacted most between 16 and 35 years of age. |
| Hassan et al., 2020 ​[25]​ | Cross-  sectional | 117 | Moshi,  Tanzania | DLQI | - DLQI mean showed moderate impairment of QoL. - QoL is influenced by the number of keloids, duration of disease and the presence of symptoms. - No significant sex predilection. |
| Reinholz et al., 2015 ​[17]​ | Cross-  sectional | 130 | Munich,  Germany | DLQI, POSAS | - Patients suffering from keloids have significantly greater effect on life quality compared to physiological scar groups (baseline) (p < 0.001) - Keloid patients scored higher in the symptoms and feelings section (p < 0.001) and daily activities section (p < 0.05) indicating higher impact on QoL. |
| Lu et al., 2021 ​[36]​ | Cross-  sectional | 553 | China | DLQI, SCL-90 | - Interpersonal relationship sensitivity, depression, and anxiety scores higher in visible scar group (p < 0.05) than invisible and normal groups. - Depression and anxiety scores higher in invisible group than normal group (p < 0.05). - Female patients’ mental health more affected than males (p < 0.05). |
| Morales-Sanchez et al., 2018 ​[38]​ | Cross-  sectional | 106 | Mexico City, Mexico | DLQI, Beck Questionnaire for  Depression | - Found minimal or small deterioration in the quality of life, most contributed by symptoms and feelings dimension of questionnaire. - The number of scars and age were directly associated with DLQI score. - Score of Beck questionnaire correlated with DLQI score. |
| Olaitan et al., 2009 ​[40]​ | Cross-  sectional | 131 | Osogbo,  Nigeria | Unspecified questionnaires | - 12.2% felt that keloids negatively affect their work. - 48.9% felt stigmatized by keloids. - 35.8% believed that keloid swelling limits their social interaction. |
| Sitaniya et al., 2022 [30] | Cross-sectional | 110 | India | Vancouver scar scale, DLQI | - Pain (p <0.001), itching (p =0.006), and restricted mobility (p <0.001) were primary causes of decreased QoL, with visibility and number of keloids not significantly affecting DLQI score. - Hyperpigmented keloids were significantly more likely to be painful (p = 0.024), which correlated with higher DLQI. - QoL correlated moderately with the severity of keloids per VSS. - History of previous treatments was associated with worsening DLQI without affecting the VSS. |
| Akakpo et al., 2024 [29] | Cross-sectional | 82 | Lomé (Togo), Africa | DLQI | - Impaired QoL was observed in 90.2% of patients. - Pruritis, aesthetic discomfort and suppuration were major factors negatively affecting QoL. |
| Motoki et al., 2019 [13] | Cross-sectional | 61 | Sao Paulo, Brazil | Body Dysmorphic Symptoms Scale (BDSS) (BDSS), Rosenberg Self-Esteem Scale | - 40% of patients with keloids surveyed screened positive for symptoms associated with body dysmorphic disorders (scored >6 on the BDSS). - The location of keloids and clinical symptoms did not significantly differ between the group of patients with body dysmorphic symptoms per the BDSS and the group that did not meet criteria per the BDSS. - Both groups—those with body dysmorphic symptoms and those without— scored low on the Rosenberg Self-Esteem Scale, suggestive of low self-esteem. |
