# Supplementary material for: Biopsychosocial impact of keloids on quality of life
Source: JAAD Rev. Author manuscript; Available in PMC 2025 Aug 1. (PMC12314879; doi:10.1016/j.jdrv.2024.08.010)
Supplement: Supplementary Figure 1 [file NIHMS2095636-supplement-Supplementary_Figure_1.pdf]

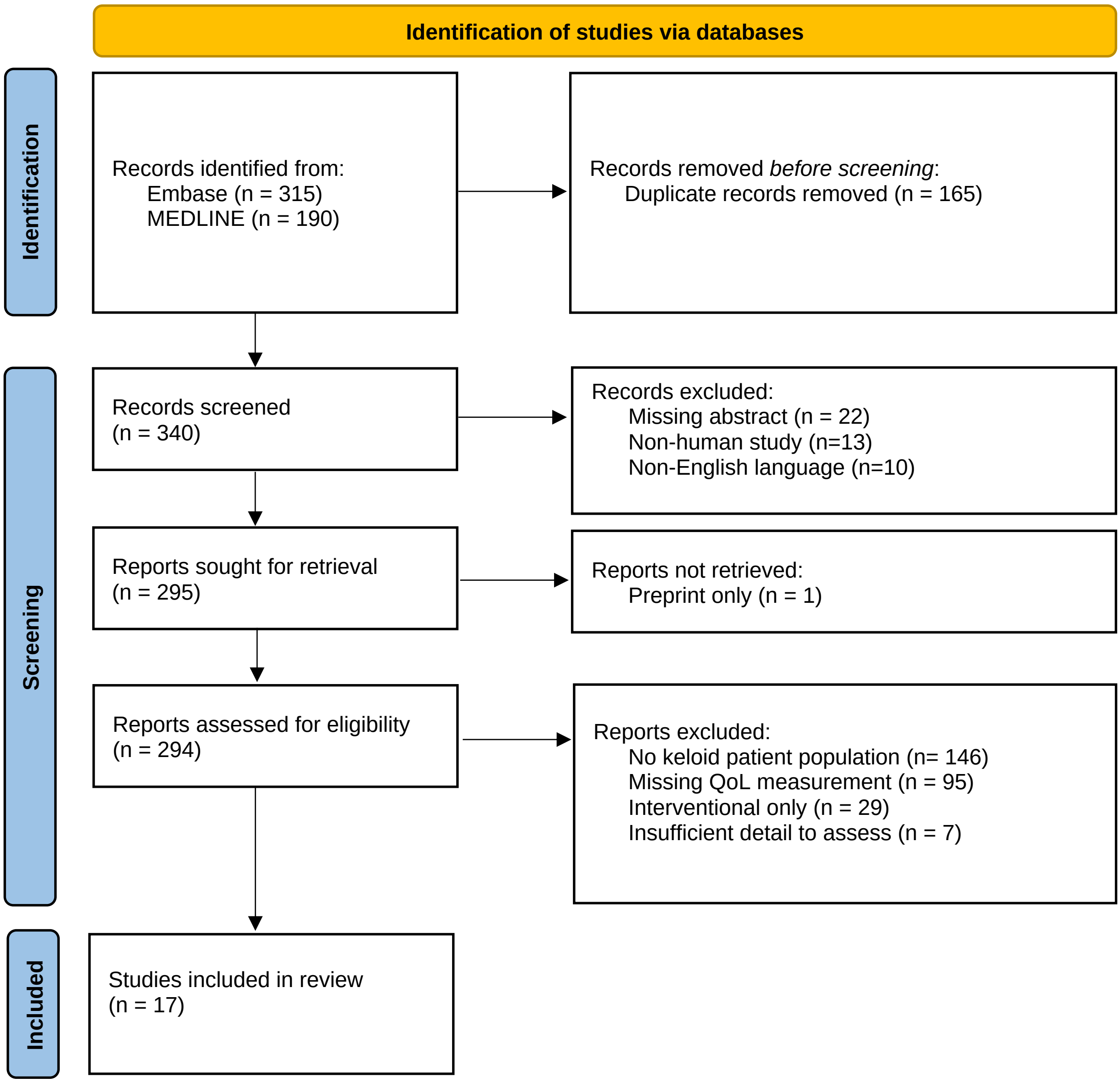

Supplementary Figure. Modified PRISMA flow diagram detailing the inclusion and exclusion criteria used to identify studies measuring keloid-associated QoL using scale- or questionnaire-based assessment tools. PRISMA diagram adapted from: Page MJ, et al. BMJ 2021;372:n71. doi: 10.1136/bmj.n71.
